# Zoomorphic Mobile Robot Development for Vertical Movement Based on the Geometrical Family Caterpillar

**DOI:** 10.1155/2022/3046116

**Published:** 2022-01-07

**Authors:** Hani Attar, Amer Tahseen Abu-Jassar, Vladyslav Yevsieiev, Vyacheslav Lyashenko, Igor Nevliudov, Ashish Kr. Luhach

**Affiliations:** ^1^Department of Energy Engineering, Zarqa University, Zarqa, Jordan; ^2^Faculty of Computer Science and Information Technology, Ajloun National University, Ajloun, Jordan; ^3^Department of Computer-Integrated Technologies, Automation and Mechatronics, Kharkiv National University of Radio Electronics, Kharkiv, Ukraine; ^4^Department of Media Systems and Technology, Kharkiv National University of Radio Electronics, Kharkiv, Ukraine; ^5^The PNG University of Technology, Lae, Papua New Guinea

## Abstract

Research in robotics is one of the promising areas in mobile robot development, which is planned to be implemented in extreme dangerous conditions of areas explored by humans. This article aims at developing and improving a prototype of zoomorphic mobile robots that are designed to repeat the existing biological objects in nature. The authors performed a detailed analysis on the structure and dynamics of the geometrical family caterpillar movement, which is passed on a practical design implemented to perform the dynamic movement on uneven vertical surfaces. Based on the obtained analysis, the design and kinematic scheme of the movement is developed. Also, the structural control scheme via the Internet technologies that allow carrying out remote control is presented in this paper, considering the dangerous mobile robot work zones. To test the recommended solutions, the authors developed detailed 3D printed models of the mobile robot constructions for the implemented hardware. The model of the mobile robot is constructed, and the control system with examples of the user program code implementations is performed. Several experiments were performed, which showed the efficiency of the achieved mobile robot for solving problems of vertical movement on uneven metal surfaces. Moreover, the obtained slow motion of the designed robot proves that the simulated robot behaves similarly to the natural behavior of caterpillar movement.

## 1. Introduction

Currently, the number of robots that developed by applying bionic principles dramatically increases, mainly the ones that are based on simulating the external nature or the kinematic functions of creatures, which are called “zoomorphic,” such as caterpillars, snakes, walking anthropomorphic robots, floating robots-fishes, flying robots-insects and birds, jumping robots-grasshoppers and kangaroos [1–4]. Compared to other types of robots, such devices simulate the most important features of the biological original, resulting in improving the passability, maneuverability, controllability, and expansion of the application areas, where the effectiveness in various operating conditions is maintained; in fact, in many scenarios, the performance even improved [1–4].

In the paper of Li et al., a group of biometric robots-fishes was developed, based on studying the energy consumption related to communal swimming in pairs, and hence, a strategy called “synchronization of vortex phases” was developed [5]. Dongfang et al. from Beijing Institute of Technology, China, developed a model of kinematics and dynamics of a multicomponent robot, as well as the equation of the joint-angle curve, expressing the thrust of each link and plotting the study's scheme, resulting in improving the existing motion control system, and consequently, proposing a new curve of motion based on the terpenoid equation [6]. Daniel et al. conducted research and developments of a particular manipulator robot that simulates the anatomy of birds. The proposed robot by Daniel et al. has a chain of motion with stimulating joints and resembles a bird's claw by grip strength.

The presented paper deeply analyzes the ornithopter-like manipulators and proposes a nonlinear controller that recognizes the limiting force of claw grip, assuming constant friction. The proposed scheme has been tested on a realistic simulator based on 5DOF-claw, low/raised leg, body, neck, and beak. Finally, the proposed design was experimentally tested in a simpler prototype 3DOF [7]. Ryan and Sarah compared the existed small-scale insect robots with the biological counterparts, where the authors provided sufficient overview research in microrobots, along with recent progress in force autonomy, mobility, and control at such a small scale. Moreover, the metrics were described to quantify the effectiveness of the investigated microrobots (e.g., speed and cost) and to quantify the path autonomy (e.g., time-dependent mass and probability of bypass) [8].

Leading robotics companies, Festo: BionicKangaroo, Bionic Cobot, Bionic ANTs, Smart Bird, Boston Dynamics, and their prototypes Spot Mini, and Wild-Cat, are engaged in Bionic Cobot research and produced models of zoomorphic robots [9–14]; the purpose of these companies is to highlight the importance of studies and development by implementing such robots in various fields of activities, such as in industry, military-space complexes, and agriculture. Indeed, the proposed work showed clearly the need to develop robotic technologies in various fields for a better technological future.

When designing and manufacturing robots to perform work in extreme conditions, much attention must be paid to study the physical prototypes, which are characterized by the motion without a continuous track and high passability in challenging road conditions. An example of such problems is described in the work of Ren et al., where the authors developed several prototype robots for testing pipes in the fields of oil and gas pumping [15]. Yang et al. developed a new creeping robot called “Hibot,” inspired by a six-legged insect at low cost, lightweight, and simple functionality. The movable shells and the wheel with legs were adapted to achieve a fixed movement of the robots with a single DC motor and without additional control. A series of experiments have shown that Hibot can craw in various environments, such as gravel and sand, and be implemented in security and disastrous investigation missions [16].

Bionic research makes it possible to create robots while maintaining the dynamic characteristics of motion, which are close to realistic prototypes of actual objects. The efficient design of robots that can simulate particular creatures requires sufficient knowledge of the creature's skeletal structure and muscular systems. The practical benefits of such expertise allow achieving smooth plastic motions that minimize the related noise and the ability to develop the required speed.

## 2. Geometrical Family Caterpillar Movement Biomechanics Analysis

Nowadays, many leading firms rely on the biomechanics of alive-style creatures in research to develop robotic systems, such as Festo Bionic and Boston Dynamics. Consequently, the proposed robot design is based on developing a mobile robot for moving along a vertical metal surface in the form of supporting beams of a bridge or similar structures. A feature of such structures is the rupture of the beams, both in the vertical and in the horizontal planes, which could be caused by the old age of the metal and/or the peculiarity of the structures. The presented paper shows that the breaks can be both microscopic and large enough such as 3 cm or more. Bridging such gaps based on classical approaches is a very difficult task, and its solution is not usually economically profitable; consequently, the implementation based on the principle of caterpillar movement allows solving such problems cheaply.

Most caterpillars have three pairs of thoracic legs (a pair on each of the chest segments) and five pairs of fake (e.g., unused for walking) abdominal legs on the III-VI and X segments of the abdomen. Abdominal paws carry small hooks located in different groups of squamous in different ways, such as in the form of a circle, longitudinal, or transverse rows. The leg consists of five joints: the pelvis, swivel, thigh, shin, and foot.

**Figure 1:**
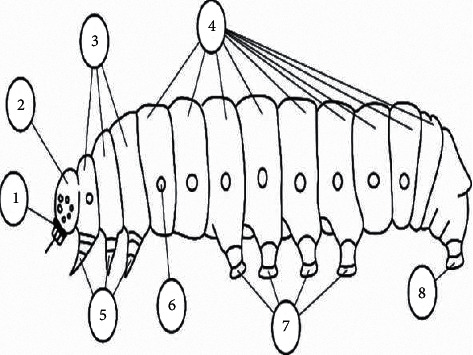
Caterpillar structure visual representation.

The thoracic legs of caterpillars are shortened compared to actual (actually walking) legs, and the function of movement is mainly abdominal legs. At the end of the thorax, a fixed claw is located that consists of different lengths and shapes. The sole is situated at the end part of the abdominal leg, which can retract, protrude, and carry the claws at its distal end. The caterpillar structure visual representation is schematically defined in Figure 1.

In the figure, 1 is the mouth device, 2 is the head, 3 is the chest segments, 4 is the abdomen segments, 5 is the chest paws, 6 is the breather (stigma), 7 is the abdominal paws (fake), and 8 is the ejector legs. As a result of this construction, the caterpillars have only thoracic legs that enable the engagement and movement, where the legs behind perform the role of the support.

Based on the above representation, it is clear that the caterpillar structure is incapable of performing the vertical movement because it has a single point of engagement to the surface, which reduces the stability of the simulated mobile robot, increases the probability of failure, and eliminates the possibility of turning around.

In 2010, through a study of geometrical family caterpillars, Simon et al. from Tufts University in the United States discovered the biomechanics of caterpillar movement based on the so-called two-body system; consequently, they designed a container by the shell of the caterpillar and the intestine that contains this container [17, 18], which is an entirely unique way of moving that does not exist in any other type of creatures. Moreover, the researchers expected to see circulating fluid flows inside the soft tissues of the caterpillars. Still, the primitive movement of the intestines of these animals prevents the fluid flow because the primitive movement is entirely independent of the rest of the body.

**Figure 2:**
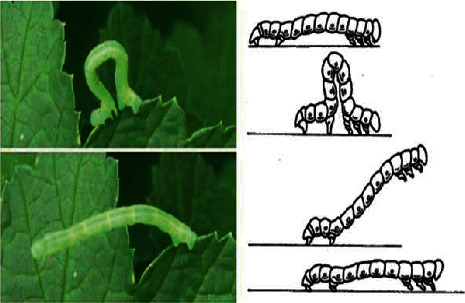
Geometrical family caterpillar movement biomechanics.

Upon further careful consideration, it became clear that with each new wave-like movement of the caterpillars, the first to move is the intestine, which moves closer to the head. The extremities of caterpillars which are called fake legs begin the movement, and the caterpillar wavy moves forward, as shown in Figure 2.

The choice of a mobile construction with a bionic principle of movement that is based on a sample of a caterpillar insect resulted in several advantages, which have high permeability and reliable contact with surfaces. To fully understand the work sequence of individual elements of the developed robot, a specific scheme of kinematic interaction is performed, which enables not only to determine the structure of the whole structure but also the nature of individual element interaction; accordingly, it is a kind of crawling description robot-caterpillar mechanisms. However, to develop a kinematic scheme of mobile robot movement based on geometrical family caterpillar biomechanics, it is necessary to analyze and choose the method of engagement that will affect the mobile robot kinematics and construction, movement control algorithms, and hardware selection.

Thus, the main task of this research is to develop a prototype of a robot that moves similarly to the movement of a caterpillar; accordingly, the analysis of the construction and kinematic scheme of mobile robot movement is practically achieved, the development of the constructive scheme is obtained, and the selection of hardware for a mobile robot including the robot control system is chosen. Ultimately, the needed software to dictate the prototype was programed and showed a smooth operation of the robot. Finally, several experimental studies were carried out over the designed robot to investigate the behavior of the robot for the suggested application.

## 3. Robots Engaging on a Vertical Surface Method Analysis

**Figure 3:**
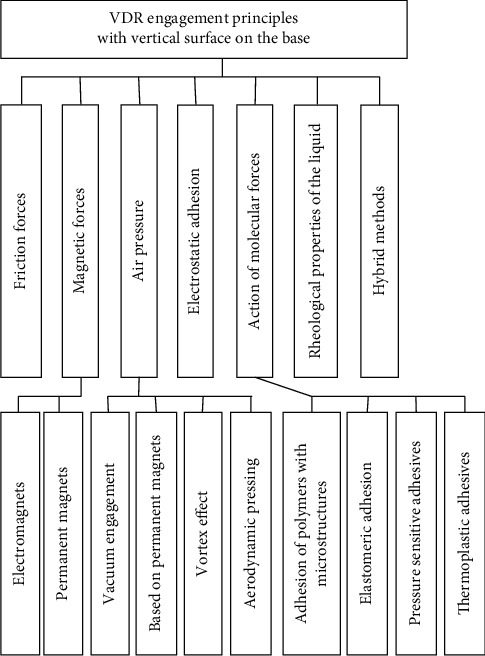
: Classification of vertical displacement robot engagement principles.

The robot surface engagement parts and methods' choice is one of the most critical criteria in the development of the vertical displacement robots, based on the tasks that are already set for the robot. Accordingly, based on the forces of nature that ensures the robot engagement to the work surface and the active and passive consumed energy (the energy consumed to move is regarded as active, and the consumed energy of holding force is regarded as passive energy) for vibration driven robots (VDRs), the engagement methods can be divided into the groups as shown in Figure 3.

Kondratenko et al. proposed a mathematical model based on the principle of electromagnetic attraction to determine the clamping force. The applied technique over robots in Kondratenko's research exploited the magnetic field theory applied over motors to enable mobile robots to move on inclined or vertical magnetic surfaces. Accordingly, the electromagnetic element is defined as a device that creates a magnetic field when an electric current passes through it [19]. Polishchuk and Oliinyk consider the work of a climber for vertical and inclined surfaces; the authors propose a new approach to improve the energy efficiency of such robots by equipping them with elastic elements [20].

Table 1 shows comparative qualitative indicators for VDR with different principles of engagement [21–25]. The movement mechanisms of VDR can also be divided into passive and active depending on whether the robot is equipped with an engine or not. Active mechanisms of VDR movement can be divided into several robotic groups: wheeled, caterpillar, walking, crawling, sliding (or frame), and hybrid.

On the one hand, Table 1 shows the qualitative indicators for VDR with different engagement principles, where –: low level, +: middle level, and ++: high level. On the other hand, Table 2 shows a summary of data that reflects the applicability and compatibility of the VDR engagement principles with the relative mechanisms of movement, collected from the existing prototypes and experimental samples of VDR [26–29].

The analysis showed that within one engagement principle, it is impossible to develop a VDR that can move on a wide range of surface types to perform the necessary work, taking into consideration that satisfying the required specifications such as low noise, high throughput, force autonomy, relatively high payload, and other essential requirements, depends on the requests of developers.

Indeed, a well understanding of robots' comparative analysis enables designers to understand that robots with vacuum gripping devices differ with insufficient reliability, passability, and the ability to move on ferromagnetic and nonferromagnetic surfaces.

Adhesive grippers are very promising but have not become widespread due to the limited number of contact cycles with surfaces and low load capacity.

To implement the layout of a zoomorphic mobile robot to move on a vertical surface, a magnetic surface-to-surface bonding method was chosen in this research work.

Though the adhesion chemical methods could be implemented for the purpose of surface-to-surface bonding, but they have several functional limits, such as moving on open metal surfaces that suffer from rust, dust deposits, pieces of dirt, water, and/or other effects; consequently, the chemical methods of adhesion are not applicable.

The magnetic type of contact devices saves the energy spent on installing the robot on the surface due to the high efficiency of the contact devices with the surface even when it is not clean or it is wet and rusty.

In the event of a power failure to the magnetic contact devices, the robot remains attached to the surface; all these advantages make this type of robot suitable for moving vertically on metal surfaces.

The crawling robot-caterpillar consists of the following main parts: 1-electromagnet 1, 2- electromagnet 2, 3-magnetic board on which the robot moves, 4-servo 1, 5-servo 2, 6-servo 3, 7-servo 4, 8-servo 5, 9-control module, and 10-transistors and resistors. Moreover, the proposed distribution parameters that affect the movement of the mobile robot on a vertical metal surface were proposed as follows: the gravity of electromagnets *F*_gea_ = 0 when the electromagnets are turned off, and *F*_gea_ > 0 when the electromagnets are turned on, *N* is the counteraction force of *F*_gea_, *F*_grav_ is the gravity force, and *F*_pull-aps_ is the force of the mobile robot movement.

The mechanism for moving the mobile robot is the compatibility of three moving parts of the torso and two statics, which are located on the top of the legs. The inner structure is built from two permanent electromagnets, where each piece of the body is equipped with its bracket enabling it to rotate, and servo, that affects the compression force of the pieces, taking into consideration that fastening of the robot to a vertical surface is carried out by means of magnetization.

The robot moves due to the sliding friction forces that occur between the support surface (metal board) and the robot magnets, in a vertical manner, in the initial stage; two magnets are turned on to allow the movement; in the next stage, one magnet goes into off mode resulting in shrinking the body of the caterpillar robot.

**Figure 4:**
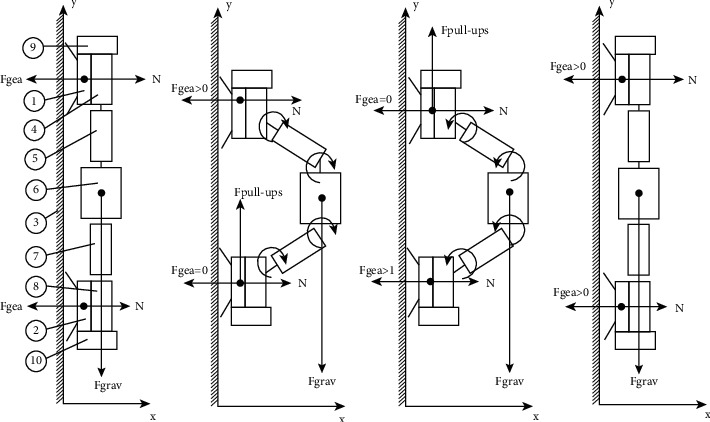
Construction and kinematic scheme of mobile robot movement.

The reduction of the robot body is due to the change of the rotation angle for the levers of the servos between all pieces of the body, bringing the rear closer to the front, and as a consequence, the robot moves up, as shown in Figure 4.

## 4. Peculiarities of Structural Scheme Development and Mobile Robot Hardware Selection

The structural scheme defines the main functional components of the robot, including the purposes and the relationships between the components.

**Figure 5:**
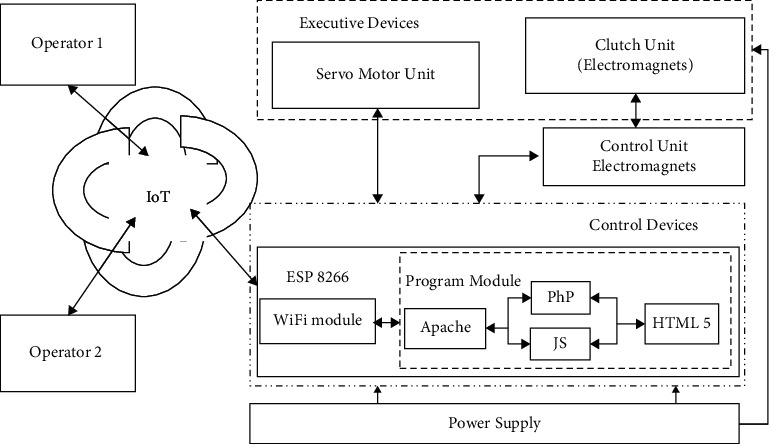
Structural scheme of a zoomorphic mobile robot.

The authors proposed and developed the structural scheme of the zoomorphic mobile robot, as shown in Figure 5, where the control system is directly implemented on the mobile robot with the possibility of remote control via Wi-Fi technology.

The crawler robot caterpillar layout control system is based on the ESP8266 WiFi module. Moreover, when choosing the microcontroller, the following restrictions were maintained: the minimum dimensions, which recommended to locate onboard a wireless connection module (Wi-Fi or Bluetooth), and choosing the appropriate set of pins to connect peripherals.

During the proposed research, the following modules were selected as follows: Arduino Nano [30], Arduino Pro Mini [31], Arduino Micro [32], and ESP8266WiFi [33]. The analysis showed that to provide a wireless connection for modules of Arduino Nano, Arduino Pro Mini, and Arduino Micro, it is required to buy an additional Wi-Fi module to connect to the board resulting in increased power consumption and connection wires, and reducing the number of pins to connect the necessary modules to control the mobile robot. Consequently, the authors suggest using the ESP8266WiFi module.

The ESP8266WiFi module is regarded as the primary unit in the proposed development because it handles and responses to the web requests besides its ability to implement the machine control algorithms and magnets to execute the movement; accordingly, it connects all parts of the crawler caterpillar robot elements and ensures the interacting between them.

The developed software module in this research includes the API programming interface provided by the developers and created an algorithm for controlling the peripherals and logic of the crawling robot-caterpillar.

Actuators consist of a block of servo motors, the levers that contribute to the maneuverability at a given angle of the required parts of the body of the crawling robot-caterpillar, and the clutch unit that fixes the robot with the surface by magnetizing the legs of the electromagnet body.

The servo-motor unit consists of 5 SG90 servo drives of model MG90S. The signals are fed to the servos directly from the WiFi ESP8266 module.

The block of electromagnets consists of two electromagnets BR 20/15 with an input voltage of 12 V and located on the edge of the body of the robot-caterpillar in terms of one on the conditional head of the robot and the other on the tail. Electromagnets are used to increase the engagement of the caterpillar when moving on ferromagnetic surfaces.

Another essential point is that to control the electromagnets, it is necessary to switch the power supplies, which is implemented by installing an electromagnet control unit based on two NPN transistors that are controlled by the WiFi module ESP8266.

The ability to control a crawling robot-caterpillar was achieved in the developed model by raising the access point on the built-in microcontroller chip and the WiFi module. As a result, the operators of the model can control the crawling robot-caterpillar by using smartphones or any other devices that support WiFi technology. It is also possible to connect several operators to the robot-caterpillar. However, the controlling process becomes available for only one device (e.g., no more than one controller at the same time).

The requests sent by operators are received by the WiFi module first and then processed and transmitted to the logic control unit using the API provided by the module developers.

### 4.1. Control Module Selection

The controller is organized based on the ESP 8266-12 microcontroller system; the following technical factors have been taken into account during the design processes: overall dimensions 24 × 16 × 2.3 mm, built-in Wi-Fi module, 32 -bit microcontroller with 160 MHz purity, and low energy consumption. So, when comparing the mentioned factors with the Arduino family (Mini, Micro, and Nano), the proposed system has a maximum purity of up to 16 MHz and does not require a separate module to work with wireless networks (Wi-Fi). As a result, the weight of the robots does not increase, which is such an important goal, because of maintaining the weight of the control system. More information about the control system specification can be found in the technical characteristics in literature [34].

**Figure 6:**
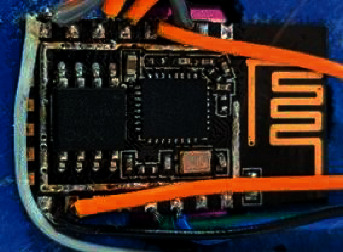
ESP 8266-12 module general view.

The board on ESP8266 is a module for WiFi communication. The implemented microcontroller has the feature of containing its SPI, UART interfaces, and the GPIO ports, which means that the module can be operated autonomously without Arduino or other boards with microcontrollers. The device can execute programs from flash memory, as the program could be run from an external SPI ROM by dynamically loading the necessary program elements. There are a vast number of varieties of ESP8266 modules. However, the proposed model developed in this work implements the module of ESP8266-12, which can work in three modes: an access point mode, client mode, or both modes simultaneously. The ESP8266-12 module is shown in Figure 6.

**Figure 7:**
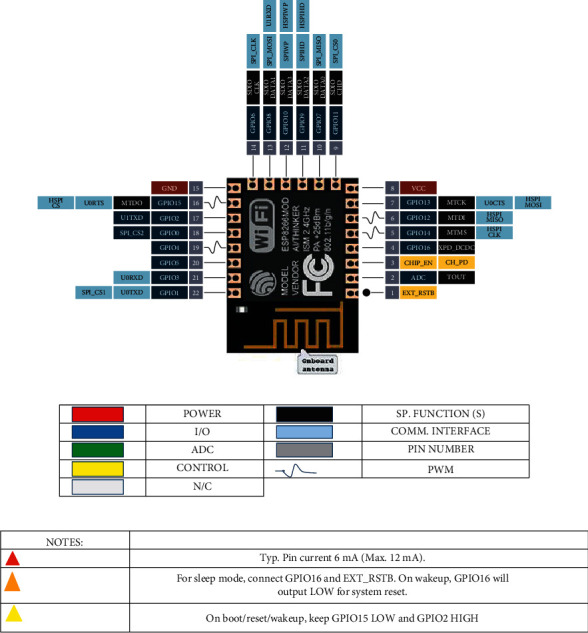
Pinout module ESP8266-12.

The ESP8266-12 module supports the IEEE802.11 b/g/n standard and a whole stack of TCP/IP protocols. Consequently, operators can use the module either as an add on to connect any device to the network or as a pinout module ESP8266-12, which is shown in Figure 7 [34].

The implemented module specifications are a built-in 32 -bit Tensilica Xtensa L106 processor with ultralow power consumption, built-in 10 -bit ADC, frequency range 2.4 GHz–2.5 GHz, supports for a clock frequency of 80 MHz, the ability to reach a maximum value of 160 MHz, supports for WiFi protocols 802.11 b/g/n with WEP, WPA, WPA2; it has 14 I/O ports, SDIO 2.0, (H) SPI, UART, I2C, I2S, IRDA, PWM, GPIO, supports for external memory up to 16 MB, required power supply from 2.2 V to 3.6 V, current consumption up to 300 mA depending on the selected mode, output power +20 dBm in 802.11 b mode, and the operating temperature range is from −40°C to 125°C.

To maintain a stable operation of ESP8266, it is required to connect a DC voltage source of 3.3 V and a maximum current of 250 mA. If the power comes from a USB-TTL converter, malti functions may occur.

#### 4.1.1. Servo Motor Selection

**Figure 8:**
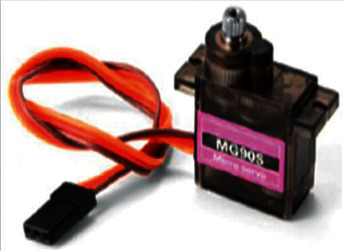
Servo model MG90S general view.

The widespread usage of servos is because of its stable operations, high resistance to interference, small size, and a wide range of speed control. The essential features of servos are the ability to increase power and provide feedback. Moreover, in the forward direction, the circuit operates as a transmitter of energy, wherein in the reverse mode, the circuit transmits the information used to improve control accuracy. The implemented servos in the proposed work are SERVO AS3103 [35], SERVO MG995 [36], SERVO S3003 [37], SERVO SG90 [38], and SERVO MG90S [39]; where all of them differ in the angle of rotation (from180° to 360°), the material of which the gearbox is made, and the overall dimensions and weight. Moreover, it is necessary when choosing servos to consider the weight restrictions of the imposed type on mobile robots in the vertical movement. As a result, SERVO MG90S was chosen, which has metal gears for extra strength and durability. The servo rotates at approximately 180° (90° in each direction) and operates in the same way the standard types operate, though it is smaller in size. The general view of the MG90S servo model is shown in Figure 8 [39].

Technical characteristics of the servo drive MG90S are as follows: speed of command working at 0.1s./60° (at 4.8 V), 0.08 s./60° (at 6 V), power supply 4.8V–6.0 V, operating temperatures from 0° to 55°, torques of 1.8 kg/cm (4.0 V) and 2.2 kg/cm (6.0 V), dead zone width of 4 microseconds, current in the motion of 50–80 mA, current in the content of 5–10 mA, rotation angle of 180°, dimensions of 22.6 mm × 12.1 mm × 22.5 mm, and weight of 12 g [39].

#### 4.1.2. Electromagnets for Engagement Selection

Based on the analysis mentioned in Tables 1 and 2, the decision of the performed motion is shown on a vertical metal surface by implementing the electromagnets. It is clear that the proposed design allows the robot to carry out the motion even on polluted surfaces, regulating the time of inclusion of electromagnets and also functioning independently from each other. Accordingly, this type of movement can be effectively implemented at different angles, which enables the mobile robot to turn when it is mounted on surfaces with only one electromagnet.

**Figure 9:**
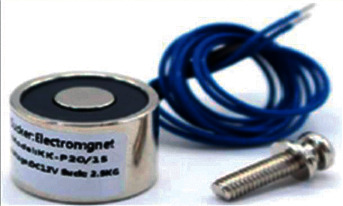
: Electromagnets BR 20/15 [41].

Based on weight and size limitations, the following types of Kinlin electromagnets were analyzed: CL-P20/15 5.0 V, 3.0 W 2.5 kg/25N [40], and CL-P20/15 12.0 V, and 3.0 W 2.5 kg/25N [41]. Despite the variety of the electromagnet types, all electromagnets consist of the following main parts: coils with conductive winding, magnetized core, and anchors to transmit the force to mechanical action. To reduce the energy losses due to the heat, the cores are made of a specific type of steel that is set in sheets. The lifting force of the electromagnet equals the force required to separate a piece of steel attached to it, which is determined by the number of turns of the coil, the current flowing through the coil, and the magnetic properties of the core. When choosing electromagnets for the robot development, it is necessary to consider that the surface of the cup-type electromagnet should be smooth (e.g., not rough) to not affect the performance efficiency. In the proposed work in this research, BR 20/15 electromagnets were selected for the implementation of the mobile robot, and its general view is presented in Figure 9.

The technical characteristics of electromagnets BR 20/15 are as follows: input voltage of 12.0 V, holding force of 2.5 kg/25N, power of 3.0 W, the material of metal, size of 20 mm × 15 mm, the ambient temperature within 130°C; and the suction thickness more than 5 cm.

### 4.2. Connection Scheme Development

**Figure 10:**
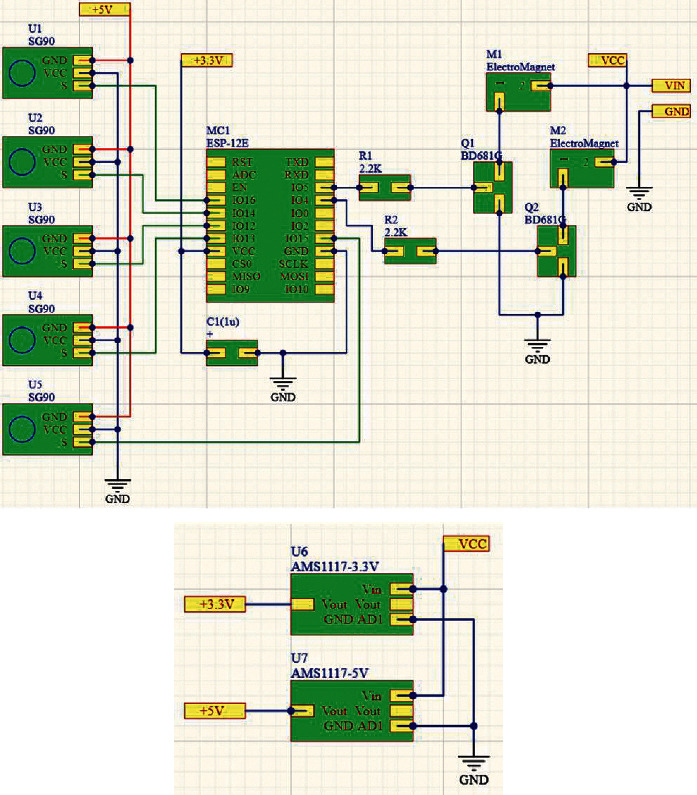
The proposed EasyEDA connection scheme.

The web-oriented environment for automating circuit development and editing the PCB topology was used to develop the connection scheme, which is EasyEDA [42]. The choice of EasyEDA scheme was based on the following advantages: cross-platform web-based electronic design automation environment includes circuit diagram editor, PCB topology editor, SPICE simulator, cloud storage, and the project management system that enables acting synchronously and remotely on the mobile robot. The connection diagram of the control system with servos and electromagnets is presented in Figure 10.

The power supply of the servo drive and the WiFi of the ESP8266 module are implemented separately based on one cotton wool voltage stabilizer AMS1117. The power supply voltage of the ESP8266 is chosen to be 3.3 V, where a voltage of 5 V is connected to supply the power for the servos.

The electromagnets are powered by the voltage of the power supply. The switching mechanism is carried out by 40.0 W transistors of the BD681 module because these types of transistors have a margin that enhances connecting more powerful electromagnets when needed. To protect the ESP8266, a 2.2 kΩ current-limiting resistor is connected to the base of the transistor.

## 5. Mobile Robot Housing Three-Dimensional Model Development

Based on the caterpillar insect structural and kinematic analysis for the developed schemes, the proposed work in this paper was represented in a developed 3D model for the investigated crawling robot-caterpillar. One of the important criteria when choosing the environment for developing a 3D model of a mobile robot is the ability to quickly export the obtained results to a 3D printing as well as the ability to use cloud storage to store the results for the teamwork on the project. The following CAD systems were considered to develop a 3D model of a mobile robot: SolidWorks [43], Fusion 360 [44], Pro/ENGINEER [45], and Tinkercad [46].

**Figure 11:**
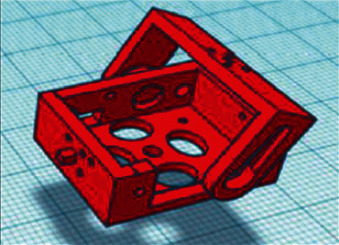
Servo mounting unit MG90S and the bracket.

**Figure 12:**
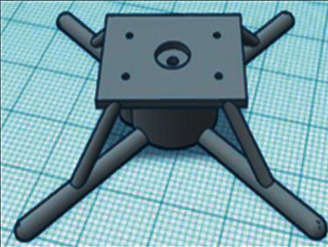
3D model in detail for fastening the electromagnets BR 20/15.

**Figure 13:**
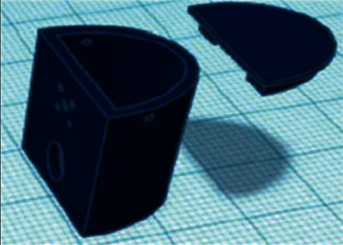
3D model of parts for peripheral storage.

The development team is opted for Tinkercad, as it is an accessible environment for developing 3D models with the ability to quickly create and 3D print, as well as expanded cloud storage. The design of the robot was divided into three stages which are as follows:

  Stage one: The development of the 3D model of brackets and blocks in which the MG90S servo driver will be located, as shown in Figure 11.
  Stage two: The development of the parts, where the electromagnets BR 20/15 3D model is located, which is shown in Figure 12.
  Stage three: The development of the parts for the peripheral storage 3D model: 40 W BD681 transistors, AMS1117 V stabilizers, and a 2.2 kΩ current-limiting resistor, which are implemented in the form of a hollow model. The general view is presented in Figure 13.

During the development of the design, parameters were laid to improve the mobility of the robot to expand the possibilities of its applications. The “barrel-shaped” robot's head and tail are justified by the ability to avoid the collision with any unpredicted obstacle (a tree branch, etc.). So, if the body was made in a square shape, then the obstacle creates resistance to the robot's movement, while the “barrel shape” avoids this effect by sliding. Moreover, the barrel shape has a better ability to install a camera positioning system when needed, or any other devices.

**Figure 14:**
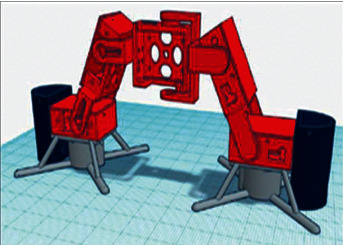
Detailed 3D model of the mobile robot housing assembly.

To ensure the proposed robot motion's stability and freedom, the supporting parts of the torso were implemented, which are based on four horizontal connections in a parallel way to the surface of the legs to provide a degree of movement freedom on the vertical surface of the metal plane. In Figure 14, a complete detailed 3D model of the proposed robot is presented.

As can be seen from Figure 14, the musculoskeletal system of the crawling robot-caterpillar was divided into three blocks as follows:

  Block one: The red block consists of five parts, where four are in the horizontal position and one (central) in the vertical position. Details of the block are intended to maintain the servo drive, where levers carry out the necessary movement of the turning corner. The presence of a vertical position for one of the parts reduces the torso to facilitate the robot's dynamic movement.
  Block two: The gray block consists of two parts that are located at the front and rear of the robot, which is designed to hold the electromagnets to assure contact with the surface of the winding friction-sliding force movement.
  Block three: The blue block is similar to the gray one, which consists of two parts. The parts are designed to keep inside all the actuators and controls of the developed robot. Accordingly, the blue box acts as a protective shell for storing essential parts because the structure is well controlled.

**Figure 15:**
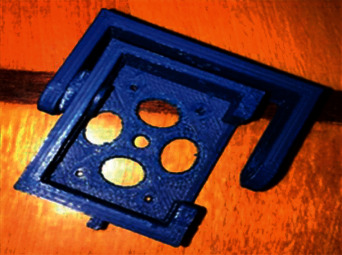
Printed MG90S servo-mounting unit and the bracket.

Using 3D printing technology based on Cpet plastic with a diameter of 1.75 mm, all 3D models with 60% fill were printed.  Figure 15 shows the printed MG90S servo-mounting unit and the bracket.

**Figure 16:**
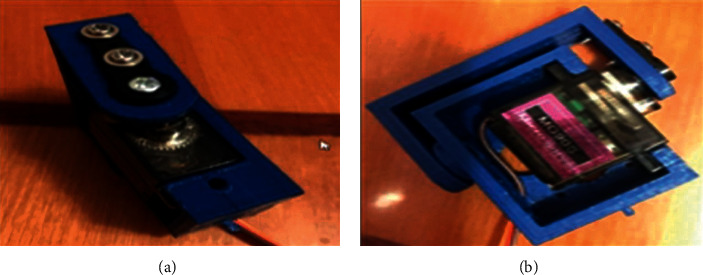
The assembled servo mounting module (a) and bracket with installed MG90S (b).

The assembled module of the servo-mounting unit and the bracket with installed MG90S is shown in Figure 16.

**Figure 17:**
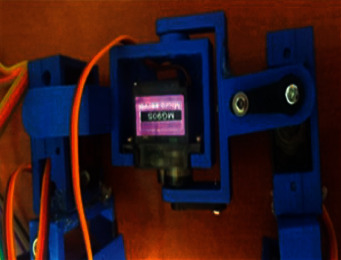
A fragment of the collected mobile robot middle sections.

A fragment of the assembled model for the middle sections of the mobile robot that enables the implementation of the reversal of the torso is shown in Figure 17.

**Figure 18:**
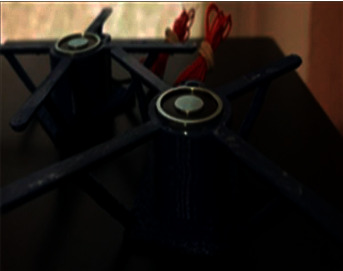
Implementation module of engagement with a metal surface using electromagnets BR 20/15.

The implementation module of engagement with a metal surface using electromagnets BR 20/15, with the established magnets, as presented in Figure 18.

### 5.1. Robot Layout Collection

The preparatory stage before compiling the layout was modeled (Figures 11–14) and printed on a 3D printer for all the necessary details of the structure (Figures 15–18). Then, after printing, the moving parts and fastening the holes of all elements were treated with sandpapers. When all the details were prepared, the process of assembling the layout began. The first stage of model assembling was to install electromagnets inside the leg and fix them with screws. In the leg, there is a groove for the wire from where it was brought. The next step was to cut one arm of each servo lever, check the combination of their sizes with the outflow located on the brackets, and refine the variety of parts with each other. Then, the levers were attached to the brackets and fixed by screws of appropriate size. Next, the brackets were connected to the parts of the torso. The details of the torso were screwed to the legs as well.

**Figure 19:**
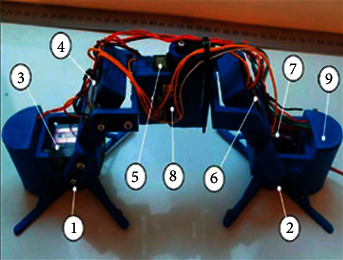
Assembled design of the proposed mobile robot-caterpillar.

The last stage of the layout was to fix all the electronics on the robot. The WiFi module ESP826612 was too large and did not fit the size of the rear part, which is designed for storage, so it was decided to attach the microcontroller to the central unit of the torso by hot-melt adhesive. Transistors, resistors, and stabilizers were installed in the head area. The wires were carefully assembled and located along with the entire structure, considering that the design bending and unbending must not interfere with the robot's movement. The completed model of the mobile robot-caterpillar is presented in Figure 19.

The layout of the mobile robot-caterpillar consists of the following main parts: 1 and 2 are the electromagnets, 3, 4, 5, 6, and 7 are the servos, 8 is the control module, and 9 is the transistors, the resistors, and the stabilizers.

## 6. Robot Motion Dynamics Development

Full attention was paid to the recently published research regarding the latest analysis and development for different mobile robots similar to the proposed one (mention dynamics type), such as the research work published by Naderi et al., that presented a semiautonomous mobile robot, which is capable of moving along a flexible environment to study the gastrointestinal tract. The result of Naderi and Najarian's work was developed by a computer model [47]. However, the proposed computer model has several disadvantages, such as the inability to move independently and the need for a humid environment because the mobile robot cannot move without humidity. Vania et al.'s research developed a model and cycle of a robotic device based on worms, which is designed as an all-wheel-drive robot manipulator with six hard links [48]. However, the proposed model and the developed prototype had a linear motion; for example, turning right and left were not considered, which significantly limits the mobility of the developed robot, as a result of which the proposed design is not suitable for the proposed task in this paper.

The next step in developing the mobile robot was the need to find the angles of rotation for the servo drive MG90S to ensure the movement. To solve this problem, a mobile robot system that has five solid blocks of structure that interact with each other is presented and located on axes that lie on one line. In the places of blocks fastening to each other, there are rotating levers of the servo drive. The repetition angle was inserted into the program, which causes a dynamic change when a set of turning angles is assumed. The two extreme blocks are controlled by electromagnets, where they perform the engagement of the structure to the surface. A shell is attached to the axes, resulting in a completely rigid body that can move in parallel. Experimentally, with the help of logical conclusions, it can be assumed that, at the initial stage, the robot is in the first step in the disengaged state and visually resembles a straight line; at this point, on each lever of the servos, the following angles are set as follows: *α*_1_ = 82°; *α*_2_ = 98°; *θ* = 0°; *α*_3_ = 98°; *α*_4_ = 82°. When moving, the robot makes a tightening movement, where the movement is defined as the second step. At this point, there is a change of four angles of servo-drive levers, one remains invariable, and then the following degrees are set as follows: *α*_1_ = 165°; *α*_2_ = 15°; *θ* = 0°; *α*_3_ = 15°; *α*_4_ = 165°. The third step is alignment, where the robot begins to unravel, stretching to its entire length. As a result, the degree sets for the servo lever are assigned to be the same as the first step.

The servo lever of the central unit remains static; it does not need to change the rotation angle when moving the robot forward or backward.

To set the rotation angles, the interval of 165°–82° is adjusted (e.g., not 180°–90°) so that the crawling robot would not step on itself when moving, so it was decided to slightly extend its base.

**Figure 20:**
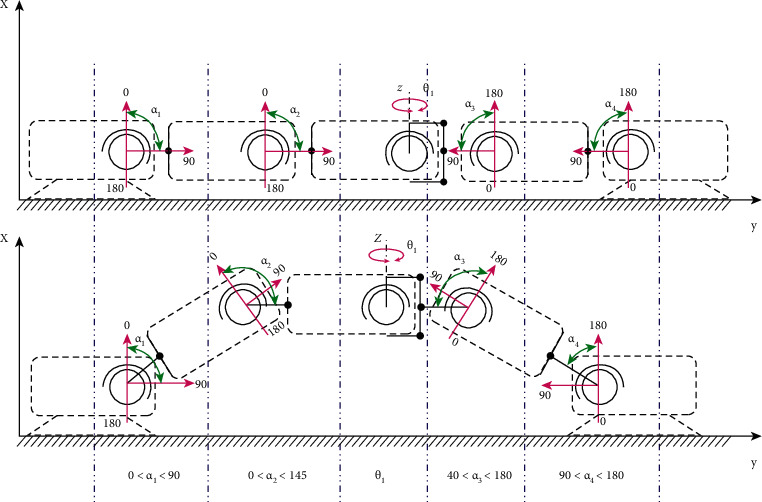
Mobile robot-caterpillar motion dynamic scheme.

Next, the situation is considered where the robot-caterpillar, after moving forward or back, has sent a request to turn right or left. To make a turn after moving in a straight line, the robot will need to repeat the first three movement steps and then go to the second step, and then, step four is performed, in which the central lever of the servo will change its position and rotate at a certain degree so that the structure can make a turn. The specified degrees will be as follows: *α*_1_ = 165°; *α*_2_ = 15°; *θ* = 75°; *α*_3_ = 15°; *α*_4_ = 165°. After completing the four steps, the robot-caterpillar will return to step two in an entire bent position of the body and then move to step one to complete the movement. For a better understanding of the robot mobility's dynamics as shown in Figure 20, a mobile robot-caterpillar motion's dynamic scheme is proposed. After completing the four steps, the robot-caterpillar will return to step two in an entire bent position of the body and then move to step one to complete the movement. Figure 20 illustrates the robot mobility's dynamic scheme for the proposed mobile robot-caterpillar.

Accordingly, when designing the proposed mobile robot, a computer model was not developed, simply because the proposed work's aim is directed to design a practical robot to be testified in actual impact over the investigated surfaces and then to evaluate the collected functional results to modify the robot accordingly. Moreover, the software to dictate the designed robot is obtained and tested working and hence included in Section 7.

## 7. Control Software for Mobile Robot Development

Based on the fact that for the mobile robot control system module ESP8266-12 is selected in the proposed work for the development of mobile robot control software, the following development environments can be used as follows: Eclipse with Arduino ESP8266 [49], Visual Studio + Visual Micro [50], and Arduino IDE [51]. It is worth noting that all the considered development environments use the Basic C and C ++ simulation languages. The Arduino IDE was chosen as an integrated development environment for Windows, MacOS, and Linux, and developed in C and C ++. Accordingly, the Arduino IDE was used to analyze the development environments and to create the design's programs to download on Arduino-compatible boards or any other board from other manufacturers.

A simple and functional development environment for creating specific software, the connection of the PC with the microcontroller implemented via the USB interface, and a code in C and C ++ are all written in an editor that has command highlighting and a spellchecker.

To program the WiFi module, it is needed to connect the official library to work with ESP8266 from the developers of the module.

**Figure 21:**
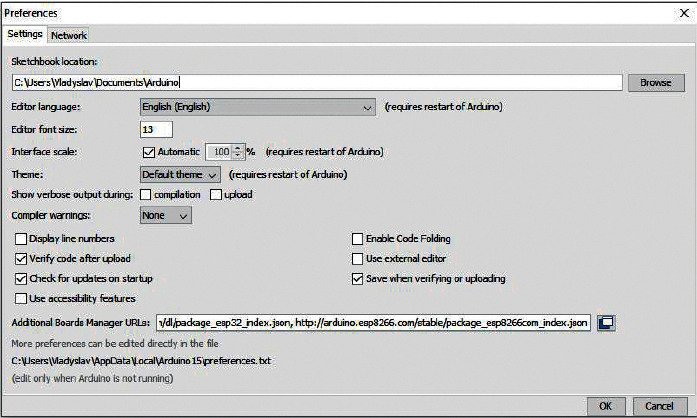
The window “Preferences” Arduino IDE for working with ESP8266.

The window “Preferences” Arduino IDE is presented in Figure 21.

To install the library for ESP8266, the following steps should be followed:

- Start the Arduino IDE, and then select “File ⟶ Settings” in the menu
- Then, insert the below link in the window of the item “Additional links for the Board Manager.”
- http://arduino.esp8266.com/stable/package_esp8266com_index.json.
- Click “OK.”

To install the board manager ESP8266, the following steps should be followed:

- From Arduino IDE menu, select “Tools ⟶ Boards ⟶ Board Manager.”
- In the text box of the “Board Manager” search, type ESP and then select “ABB Aurora PV inverter library for Arduino, esp8266 and esp32,” and click “Install.”
- When the installation is complete, click the “Close” button.

**Figure 22:**
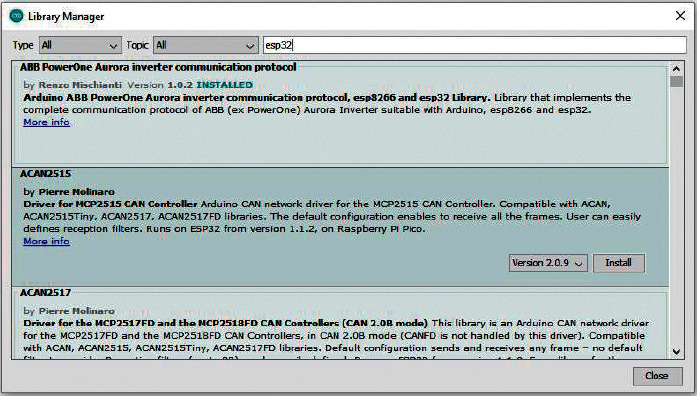
The Arduino IDE Board Manager window.

The Arduino IDE Board Manager window is shown in Figure 22.

After the performed manipulations, it will be possible to connect the necessary libraries to the executable code using the #include command, as well as view examples for this WiFi module.

### 7.1. Development of a Mobile Robot Control Program

Based on the selected Arduino IDE development environment, implementation of the writing of a control program is performed in the form of a sequence of the following steps:

At the first step, the necessary libraries are connected as follows:

- ESP8266WiFi.h, to connect ESP8266 to Wi-Fi
- WiFiClient.h, to create a client with an IP address through the selected port
- ESP8266WebServer.h, to create a standalone Web server
- Servo.h, for servo control.

Define preprocessor directives:

  #ifndef APSSID
  #define APSSID “Guslay” /^*∗*^network name^*∗*^/
  #define APPSK “12345678”/^*∗*^password^*∗*^/
  #endif
  The next step is to describe the data:
  const char ^*∗*^ssid = APSSID;
  const char ^*∗*^password = APPSK;
  int HFSPin = 12; /^*∗*^pin connection of 1 servomotor ^*∗*^/
  int VFSPin = 13; /^*∗*^pin connection of 2 servomotor ^*∗*^/
  int SPin = 14; /^*∗*^pin connection of 3 servomotor ^*∗*^/
  int VRSPin = 15; /^*∗*^pin connection of 4 servomotor ^*∗*^/
  int HRSPin = 16; /^*∗*^pin connection of 5 servomotor ^*∗*^/
  int FrontMagnet = 5; /^*∗*^pin front magnet connection ^*∗*^/
  int RearMagnet = 4; /^*∗*^pin rear magnet connection ^*∗*^/
  int servoCorrectValue [5] = {0,3,−12,6,0}; /^*∗*^pin creating an array ^*∗*^/.
  int angle = 90, steerAngle = 0, stapStage = 0,
  steerStage = 0; /^*∗*^ description of angles name and their meanings ^*∗*^/
  bool startMove = false;
  bool revers = false;
  bool stopMove = false;
  bool startSteer = false;
  bool stopSteer = false;
  bool steerLeft = false;
  bool desebleMagnets = false;
  ESP8266WebServer server(80); /^*∗*^ set the port number for the Web server ^*∗*^/
  Servo horizontalFrontServo; /^*∗*^ determination of 1 servomotor ^*∗*^/
  Servo verticalFrontServo; /^*∗*^ determination of 2 servomotor ^*∗*^/
  Servo steerServo; /^*∗*^ determination of 3 servomotor ^*∗*^/
  Servo verticalRearServo; /^*∗*^ determination of 4 servomotor ^*∗*^/
  Servo horizontalRearServo; /^*∗*^ determination of 5 servomotor ^*∗*^/

Next, using const String webSite in quotation marks, the HML code of the page is inserted with the control buttons of the mobile robot; the syntax of the record is as follows:

  const String webSite = “<!DOCTYPE html><html lang = \“en\”><head><meta charset = \“UTF-8\”><meta http-equiv = \“X-UA-Compatible\” content = \“IE = edge\”><meta name = \“viewport\” content = \“width = device-width,initial-scale = 1.0\”><title>Controller</title> ……</body></html>”;

Letus create a procedure for receiving a message from the server to control servomotors and command functions for control.

  void handleRoot() {
  server.send(200, “text/html”, webSite);
  void StartMoveForward(){ /^*∗*^ moving forward ^*∗*^/
  server.send(204);
  startMove = true;
  revers = false;
  stopMove = false;
  }
  void StopMoveForward(){ /^*∗*^ stop moving ^*∗*^/
  server.send(204);
  startMove = false;
  revers = false;
  stopMove = true;
  }

Based on the following functions StartMoveForward and StopMoveForward, it is necessary to develop functions for moving backwards, left, and right and disconnecting the electromagnets.

The next step is to describe the void setup ()function to initialize the variables and commands that will be executed throughout the mobile robot.

  horizontalFrontServo.attach(HFSPin); /^*∗*^ connection of 1 servomotor ^*∗*^/
  verticalFrontServo.attach(VFSPin); /^*∗*^ connection of 2 servomotor ^*∗*^/
  steerServo.attach(SPin); /^*∗*^ connection of 3 servomotor ^*∗*^/
  verticalRearServo.attach(VRSPin); /^*∗*^ connection of 4 servomotor ^*∗*^/
  horizontalRearServo.attach(HRSPin); /^*∗*^ connection of 5 servomotor ^*∗*^/
  Serial.begin(115200); /^*∗*^ port setting ^*∗*^/
  Serial.println();
  Serial.print(“Configuring access point…”);
  WiFi.softAP(ssid, password); /^*∗*^ setting up an IP address connection ^*∗*^/
  IPAddress myIP = WiFi.softAPIP();
  Serial.print(“AP IP address: ”);
  Serial.println(myIP);

To process incoming HTTP requests, it is needed to specify the code to execute when calling a specific URL. To do so, a method that takes two parameters is used. The first parameter is the URL path, and the second parameter is the name of the function being performed when navigating from the URL.

  server.on(“/”, handleRoot);
  server.on(“/forward-start”, StartMoveForward);
  server.on(“/forward-finish”, StopMoveForward);
  server.on(“/back-start”, StartMoveBack);
  server.on(“/back-finish”, StopMoveBack);
  server.on(“/right-start”, StartMoveRight);
  server.on(“/right-finish”, StopMoveRight);
  server.on(“/left-start”, StartMoveLeft);
  server.on(“/left-finish”, StopMoveLeft);
  server.on(“/disable-magnets”, DisableMagnets);
  server.begin();
  Next, it is needed to adjust the angles of the servomotors
  horizontalFrontServo.write(90);
  verticalFrontServo.write(90);
  steerServo.write(90+servoCorrectValue [2]);
  verticalRearServo.write(90);
  horizontalRearServo.write(90);

The next step is to write the function void loop (). The authors proposed the following implementation of this function to control a mobile robot.

  void loop() {
  server.handleClient();
  if(disableMagnets){
  disableMagnetDelay--;
  if(disableMagnetDelay ≤ 0)
  {
  Magnetization of a mobile robot to a vertical surface:
  disableMagnetDelay = 500; /^*∗*^ delay ^*∗*^/
  SetRearMagnetState(true); /^*∗*^ magnet 1 on^*∗*^/
  SetFrontMagnetState(true); /^*∗*^ magnet 2 on ^*∗*^/
  disableMagnets = false; /^*∗*^ the magnet mute button is deactivated ^*∗*^/
  }
  delay(10);
  }

A fragment of the mobile robot movement program code is as follows:

  if((startSteer || stopSteer) && !(startMove || stopMove)&&!disableMagnets){
  if(steerStage = = 1 && stopSteer){
  steerStage = 2;
  }
  Serial.print(“Stear: ”);
  Serial.println(steerAngle);
  Serial.println(steerStage +“: ” + stopSteer);
  switch(steerStage){
  case 0:
  horizontalFrontServo.write((180-angle)+servoCorrectValue[0]);
  steerServo.write(90+servoCorrectValue [2]);
  horizontalRearServo.write((180-angle)+servoCorrectValue [4]);
  SetRearMagnetState(true);
  delay(100+moveSpeed);
  SetFrontMagnetState(false);
  if(!startSteer && stopSteer){
  stopSteer = false;
  SetRearMagnetState(true);
  SetFrontMagnetState(true);
  }
  steerStage++;
  break;
  case 1:
  steerAngle++;
  horizontalFrontServo.write((180-angle) + servoCorrectValue[0]-footUp);
  steerServo.write(90 + servoCorrectValue [2] + steerAngle ^*∗*^ (steerLeft?-1:1));
  horizontalRearServo.write((180-angle) + servoCorrectValue [4]);
  if(steerAngle≥75)
  steerStage++;
  delay(moveSpeed);
  break;
  case 2:
  horizontalFrontServo.write((180-angle) + servoCorrectValue[0]);
  //steerServo.write(90+servoCorrectValue [2]);
  horizontalRearServo.write((180-angle) + servoCorrectValue [4]);
  steerStage++;
  SetFrontMagnetState(true);
  delay(100 + moveSpeed);
  SetRearMagnetState(false);
  break;
  case 3:
  steerAngle--;
  horizontalFrontServo.write((180-angle) + servoCorrectValue[0]);
  steerServo.write(90+servoCorrectValue [2] + steerAngle ^*∗*^ (steerLeft?-1:1));
  horizontalRearServo.write((180-angle)+servoCorrectValue [4]-footUp);
  if(steerAngle≤0)
  steerStage = 0;
  delay(moveSpeed);
  break;
  }

The developed mobile robot remote control system gives several advantages, which are as follows:

- Control program is mounted on the mobile robot, which is allowed if necessary to switch between operators;
- Mobile robot control can be performed wirelessly for a distance of 80-100 meters in the open space, and when using a narrowly directed Wi-Fi antenna, the distance could reach up to 230 m. Consequently, the operator is allowed to be at a relatively far length from the danger zone, wherein in some applications, 230 m is regarded as remarkably enough.
- It has the ability to use computer vision systems, identification, and decision-making systems, with a sharp break in communication with the mobile robot.

To improve the communication range for the robot, a network of robot-caterpillar can add more features of the developed robot by taking more advantage of other published works, such as designing a net of robots that can communicate wirelessly by applying the hybrid network proposed in [52] based on the co-operation between communicating nodes [53, 54], as a suitable solution for the lossy channel environment, which is expected in extreme conditions.

### 7.2. Remote Robot Control Program Code

The following programming languages HTML +, CSS +, and JS were used to implement the web interface for mobile robot control. The following is an example of a control interface implementation that is then imported into const String webSite.

  <!DOCTYPE html>
  <html lang = “en”>
  <head>
  <meta charset = “UTF-8”>
  <meta http-equiv = “X-UA-Compatible” content = “IE = edge”>
  <meta name = “viewport” content = “width = device-width, initial-scale = 1.0”>
  <title>Controller</title>
  <style>
  html {
  height: 100%;
  --width-btn: 100px;
  }
  body {
  height: 100%;
  margin: 0;
  background-color: #b6b4a7;
  display: flex;
  flex-direction: column;
  justify-content: center;
  align-items: center;
  }
  The description of the control buttons:
  button:active span {
  transform: translateY(0);
  }
  button svg {
  height: calc(var(--width-btn)/5 ^*∗*^ 3);
  width: calc(var(--width-btn)/5 ^*∗*^ 3);
  }
  ……
  </style>
  </head>

Next in the tag <body>, the description of the work with the buttons to control the movement of the mobile robot and a fragment of the implementation of the movement of the robot forward (up) are presented below:

  <button id = “btnMoveForward”>
  <span>
  <svg width = “12” height = “8” viewBox = “0 0 12 8”>
  <g transform = “rotate(-360 517 942)” id = “arrow” fill = “currentColor”>
  <path d = “M10.589 0L6 4.58 1.41 0 0 1.41l6 6.001 5.999-6.001z” />
  </g>
  </svg>
  </span>
  </button>

Based on the proposed example, it is necessary to implement the buttons to move left, right, and back (down), as well as the button to turn off the magnets.

**Figure 23:**
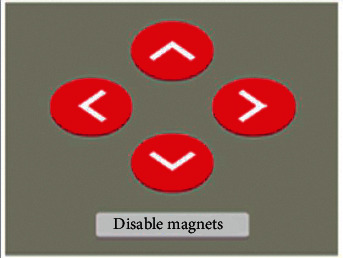
Web control interface for mobile robot.

The result of the development of the web control interface is presented in Figure 23.

### 7.3. Experimental Studies

**Figure 24:**
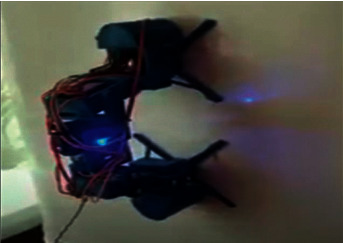
Actual movement test for a mobile robot at a 90-degree vertical metal surface.

In this section, actual and practical tests were performed over the developed model to confirm the correct response of the robot for the received orders. Indeed, the correct decisions made by developing the robot on the kinematics and dynamics movement on vertical metal surfaces have been confirmed. The first test was aimed to analyze the speed of mobile robot movement depending on the angle of the surface in the range from 45° to 110°.  Figure 24 shows a fragment of the test of the mobile robot movement at a 90° vertical metal surface.

**Figure 25:**
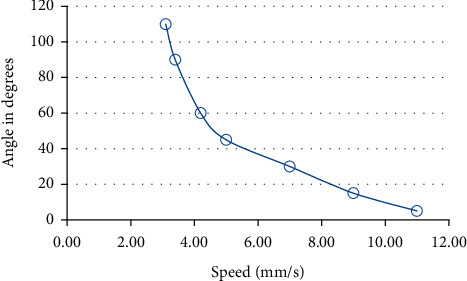
The results of the experiment.

The results of the experiment are presented in Figure 25, which illustrates that, at inclinations of 110°, i.e., the mobile robot is in a hanging position relative to the horizon, and speed drops to a minimum of 3 mm per second, this result is due to the transfer of the gravity center, which increases the mass and therefore reduces the force of engagement to the metal surface. Though the inverse relation between the gravity and the speed of motion is regarded as a disadvantage, it is solid evidence that the designed robot does simulate the caterpillar precisely because the drop in the speed at such an angle is what happens for the actual caterpillar, i.e., the tested robot shows the natural behavior of the caterpillar.

When conducting an experiment on the horizontal movement of the mobile robot, a stable result was obtained, which is 11-12 mm per second. This result can be improved by increasing the speed of the MG90S servo motor, but taking into account the disadvantages of the servo-motor type, which is the accumulation of error at bending angles; however, this problem can be easily solved by implementing more expensive servo motors or implementing stepper motors.

Moreover, in the proposed paper, the practical testing shows that the robot overcomes structural breaks up to 10 cm, regardless of the vertical or horizontal types of movement. Moreover, the overall small dimensions of the proposed robot make it to use in hard-to-reach narrow places (the maximum width of the mobile robot of 7 cm, height of 5 cm, stretched length of 25 cm, and the length during moving of 25 cm). It is also possible to switch between horizontal and vertical movement without changing the design. Moreover, the proposed robot is experimentally developed to enhance its capabilities. Accordingly, the robot can be upgraded and improved to the requirements and tasks that need to be solved; in fact, developing the designed robot can even be extended to nano-robots that enable applying such robotics in medical research and applications such as a supportive tool in operations and for the diagnosis of human diseases in the internal organs.

## 8. Conclusion

The proposed work in this paper is aimed at designing and developing a mobile robot-caterpillar that simulates the natural behavior of the actual insect, based on the geometrical family caterpillar movement kinematics. Indeed, the caterpillar has several advantages that enable it to work under extreme conditions and with different types of services, which is why authors decided to simulate the caterpillar in particular. The obtained results were collected from the implemented hardware of the robot-caterpillar, which shows clearly that the developed robot simulates the natural behavior of the caterpillar. Moreover, to improve the designed robot, it is just needed to install higher specifications of the robot components.

Based on the above, the obtained results are regarded as interested for beginners and specialists in this field.

The developed 3D models and design solutions that allow improving experimental layouts and the implemented control system are regarded as universal and that could be used for most mobile robot types.

The future work is planned to be directed to improve the obtained robot by implementing higher parameters for the installed parts and to solve the problem of the proposed robot's slow motion.

A promising direction is also the development of specialized robotic systems that can be used in medical research. For the above-mentioned purposes, it is advisable to upgrade the prototype of the proposed robot, considering the specific parameters of medical research. At the same time, it should be noted that the proposed design features of a mobile robot can be the basis for the development of mobile medical robots.

## Figures and Tables

**Table 1:** Qualitative indicators for VDR with different engagement principles.

Principles of adhesion and VDR parameters		Material parameters			Noise level
		Different materials	Dirty surfaces	Uneven surfaces	
Friction force		+	++	++	–
Magnetic	Electromagnets	–	+	+	–
	Permanent magnets	–	+	+	–
Air pressure	Active vacuum	++	+	+	++
	Passive vacuum	++	+	+	–
	Vortex effect	++	+	++	++
	Bernoulli effect	++	++	++	++
	Aerodynamics	++	++	++	++
Electrostatic adhesion		++	++	++	–
Dry adhesion	Polymers with microstructures	++	–	+	–
	Elastomers	++	–	+	–
Moisture adhesion	Glue	++	–	–	–
	Thermoplastics	++	–	–	–

**Table 2:** Application and compatibility of engagement methods and VDR movement mechanisms.

Principles of adhesion and VDR parameters		Material parameters			Noise level
		Different materials	Dirty surfaces	Uneven surfaces	
Friction force		+	++	++	–
Magnetic	Electromagnets	–	+	+	–
	Permanent magnets	–	+	+	–
Air pressure	Active vacuum	++	+	+	++
	Passive vacuum	++	+	+	–
	Vortex effect	++	+	++	++
	Bernoulli effect	++	++	++	++
	Aerodynamics	++	++	++	++
Electrostatic adhesion		++	++	++	–
Dry adhesion	Polymers with microstructures	++	–	+	–
	Elastomers	++	–	+	–
Moisture adhesion	Glue	++	–	–	–
	Thermoplastics	++	–	–	–

## Data Availability

The data that support the findings of this study are available from the corresponding author upon reasonable request.

## References

[1] Kim S., Kim Y., Kim M., Song J., Yun D. (2019). Development of quadrupedal robot mimicking the motion of snake. *Journal of Korea Robotics Society*.

[2] Wang K., Marsh D., Saputra R. P. Design and control of SLIDER: an ultra-lightweight, knee-less, low-cost bipedal walking robot.

[3] Kajita S., Hirukawa H., Harada K., Yokoi K. (2014). Introduction to Humanoid Robotics. *STAR*.

[4] Chi Z., Wei Z., Liping M., Zhiqing W. (2020). Biologically inspired jumping robots: a comprehensive review. *Robotics and Autonomous Systems*.

[5] Li L., Nagy M., Graving J. M., Bak-Coleman J., Xie G., Couzin I. D. (2020). Vortex phase matching as a strategy for schooling in robots and in fish. *Nature Communications*.

[6] Dongfang L., Chao W., Hongbin D., Yiran W. (2020). Motion planning algorithm of a multi-joint snake-like robot based on improved serpenoid curve. *IEEE Access*.

[7] Daniel F., José A., Anibal O. (2021). Control aware of limitations of manipulators with claw for aerial robots imitating bird’s skeleton. *IEEE Robotics and Automation*.

[8] Ryan P., Sarah B. (2019). Toward autonomy in sub-gram terrestrial robots. *Annual Review of Control, Robotics, and Autonomous Systems*.

[9] Bionic K. (2014). Energy-efficient jump kinematics based on a natural model, [Type of medium]. https://www.festo.com/group/ru/cms/10219.htm.

[10] https://www.festo.com/group/en/cms/12746.htm.

[11] https://www.festo.com/group/ru/cms/10157.htm.

[12] https://www.festo.com/group/ru/cms/10238.htm.

[13] https://www.bostondynamics.com/spot.

[14] https://www.bostondynamics.com/legacy.

[15] Ren T., Zhang Y., Li Y., Chen Y., Liu Q. (2019). Driving mechanisms, motion, and mechanics of screw drive in-pipe robots: a review. *Applied Sciences*.

[16] Yang M., Kang R., Chen Y. A highly mobile crawling robot inspired by hexapod insects.

[17] Simon M. A., Fusillo S. J., Colman K., Trimmer B. A. (2010). Motor patterns associated with crawling in a soft-bodied arthropod. *Journal of Experimental Biology*.

[18] Simon M. A., Woods W. A., Serebrenik Y. V. (2010). Visceral-locomotory pistoning in crawling caterpillars. *Current Biology*.

[19] Kondratenko Y., Zaporozhets Y., Rudolph J., Gerasin O., Topalov A., Kozlov O. Features of clamping electromagnets using in wheel mobile robots and modeling of their interaction with ferromagnetic plate.

[20] Polishchuk M. N., Oliinyk V. V. Dynamic model of a stepping robot for arbitrarily oriented surfaces.

[21] Lee C., Sharif M., Vinayagan S., Othman W., Alhady S., Wahab A. (2021). Design and development of a quadruped shuffling mobile robot. *Journal of Physics: Conference Series*.

[22] Polishchuk M., Suyazov M., Opashnyansky M. Study on numerical analysis of dynamic parameters of mobile walking robot. *Journal of Mechanical Engineering and Sciences*.

[23] Campion G., Bastin G., Dandrea-Novel B. (1996). Structural properties and classification of kinematic and dynamic models of wheeled mobile robots. *IEEE Transactions on Robotics and Automation*.

[24] Alexander J. C., Maddocks J. H. (1990). On the kinematics of wheeled mobile robots. *Autonomous Robot Vehicles*.

[25] Indiveri G. (2009). Swedish wheeled omnidirectional mobile robots: kinematics analysis and control. *IEEE Transactions on Robotics*.

[26] Yue T., Bloomfield-Gadêlha H., Rossiter M. Friction-driven three-foot robot inspired by snail movement.

[27] Anirban C., Shahid A., Subhasis B. Earthworm like modular robot using active surface gripping mechanism for peristaltic locomotion.

[28] Zhu Y., Fei Y., Xu H. (2018). Stability analysis of a wheel-track-leg hybrid mobile robot. *Journal of Intelligent and Robotic Systems*.

[29] Li Y., Yao Y.-a., He Y. (2018). Design and analysis of a multi-mode mobile robot based on a parallel mechanism with branch variation. *Mechanism and Machine Theory*.

[30] https://store.arduino.cc/arduino-nano.

[31] https://store.arduino.cc/arduino-pro-mini.

[32] https://store.arduino.cc/arduino-micro.

[33] https://www.espressif.com/sites/default/files/documentation/0a-esp8266ex_datasheet_en.pdf.

[34] https://www.instructables.com/NodeMCU-ESP8266-Details-and-Pinout/.

[35] https://www.autobotic.com.my/servo/servo-as3103.

[36] https://datasheetspdf.com/pdf/839879/ETC/MG995/1.

[37] https://servodatabase.com/servo/futaba/s3003.

[38] https://datasheetspdf.com/pdf/791970/TowerPro/SG90/1.

[39] https://datasheetspdf.com/pdf/1106582/ETC/MG90S/1.

[40] https://en.aliradar.com/item/32808703087-20-15-mm-suction-2.5kg-25n-dc-5v-12v-24v-mini-solenoid-electromagnet-electric-lifting-electro-magnet-strong-holder-cup-diy-12-v.

[41] https://robu.in/product/electric-sucker-electromagnet-kk-p20-15-12v/.

[42] https://easyeda.com/en.

[43] https://www.solidworks.com/lp/3dexperience-solidworks-offers.

[44] https://www.autodesk.com/products/fusion-360/overview.

[45] https://www.ptc.com/en/products/creo/pro-engineer.

[46] https://www.tinkercad.com/.

[47] Naderi N., Najarian S., Hosseinali A., Karevan H. (2013). Modeling and dynamic analysis of the worm-like part of an innovative robot applicable in colonoscopy. *International Journal of Medical Robotics and Computer Assisted Surgery*.

[48] Vania L., Ivan S., David C., Alejandro G., Misael M., Isaac C. (2020). Hybrid state constraint adaptive disturbance rejection controller for a mobile worm bio-inspired robot. *Mathematical and Computational Applications*.

[49] https://iot.eclipse.org/community/resources/articles/2015-06-08-esp8266-eclipse/.

[50] https://www.visualmicro.com/.

[51] https://www.arduino.cc/en/software.

[52] Attar H. H., Solyman A. A. A., Alrosan A., Chakraborty C., Khosravi M. R. (2021). Deterministic cooperative hybrid ring-mesh network coding for big data transmission over lossy channels in 5G networks. *EURASIP Journal on Wireless Communications and Networking*.

[53] Attar H., Stankovic L., Stankovic V. (2012). Cooperative network-coding system for wireless sensor networks. *IET Communications*.

[54] Attar H., Vukobratovic D., Stankovic L., Stankovic V. Performance analysis of node cooperation with network coding in wireless sensor networks.

